# The Nabidae (Insecta, Hemiptera, Heteroptera) of Argentina

**DOI:** 10.3897/zookeys.333.5084

**Published:** 2013-09-20

**Authors:** Marcela Cornelis, María C. Coscarón

**Affiliations:** 1 Universidad Nacional de La Pampa. Facultad de Ciencias Exactas y Naturales. Uruguay 151 L6300CLB, Santa Rosa, La Pampa. Argentina; 2 Universidad Nacional de La Plata. Facultad de Ciencias Naturales y Museo. División Entomología. Paseo del Bosque s/n 1900, La Plata, Buenos Aires. Argentina

**Keywords:** Nabidae, key, Argentina, taxonomy, distribution

## Abstract

In Argentina, five genera and 14 species are recorded in the subfamilies Prostemmatinae and Nabinae: *Hoplistoscelis sordidus* Reuter, *Lasiomerus constrictus* Champion, *Metatropiphorus alvarengai* Reuter, *Nabis argentinus* Meyer-Dür, *Nabis (Tropiconabis) capsiformis* Germar, *Nabis faminei* Stål, *Nabis paranensis* Harris, *Nabis punctipennis* Blanchard, *Nabis roripes* Stål, *Nabis setricus* Harris, *Nabis tandilensis* Berg, *Pagasa (Pagasa) costalis* Reuter, *Pagasa (Lampropagasa) fuscipennis* Reuter and *Pagasa (Pagasa) signatipennis* Reuter.

## Introduction

The Nabidae, often called damsel bugs, are a small group of predatory insects of various shapes and colours, ranging from 5 to 15 mm.

In the Neotropical catalogue, Volpi and Coscarón (2010) and Coscarón and Volpi (2013) provided a summary of the classification of the group and an exhaustive introduction to the literature. Two subfamilies, Nabinae and Prostemmatinae, comprising 11 genera and 83 species are recognized in the Neotropical Region and in Argentina four genera and 13 species (Coscarón, submitted). Although all of them are predators, some have been observed feeding on plants (Ridgway and Jones 1968, Stoner 1972, Kiman and Yergan 1985). The members of Nabinae feed on many different small arthropods including aphids, leafhoppers, larvae and adults of Hemiptera, thrips, flies, caterpillars, beetle larvae, eggs of insects, small spiders and acari, whereas those of Prostemmatinae appear to prey exclusively on other Heteroptera, especially Lygaeoidea (Pericart 1987, Lattin 1989). The predaceous habit, together with the widespread occurrence of some species in agroecosystems, has attracted the attention of entomologists (Lattin 1989). Although all known species are terrestrial, some have been found in moist areas on the ground or at the edge of streams, ponds, and marshes (both fresh and saline) (Lattin 1966, 1989, Pericart 1987).

Argentina, the geographical area considered in this paper, lies in the Neotropical Region. The country covers an area of 2,791,810 km^2^ and is bordered by Uruguay, Brazil, Paraguay, Bolivia, and Chile. Approximately 75% of the country is occupied by arid and semiarid areas, but rainforests are also present in the northeastern part of the country, for example the Yungas and Paranaense regions.

Knowledge of the South American fauna is poor, especially in relation to the taxa of economic importance, and no comprehensive keys for identification of the species living in the region have previously been published.

The objective of this paper is to provide an illustrated key of the genera of Nabidae from Argentina, including a diagnosis, geographical distribution, list of species for each genus, and a redescription when necessary.

## Materials and methods

The specimens examined belong to the collections of the Museo de Ciencias Naturales de La Plata (MLP), La Plata, Buenos Aires, Argentina (http://www.fcnym.unlp.edu.ar/abamuse.html), the American Museum of Natural History (AMNH), New York, USA (http://www.amnh.org/) and the Swedish Museum of Natural History (NHRS), Stockholm, Sweden (http://www.nrm.se/).

Observations were made with a Leica MZ95 stereoscope and measurements with a micrometer eyepiece and expressed in millimeters (mm). The male genitalia were dissected by removing the pygophore from the abdomen with a pair of forceps, and the female genitalia by cutting the genital plates with 12 cm dental standard straight scissors. Genitalia from both sexes were cleared in a KOH solution for 24 hours. All dissected structures were stained with methylene blue and photographed in glycerin. Images of adults and genitalia were taken with a digital camera (Kodak 3.1 megapixels) and a magnifying Wild M-Stereomicroscope. The terminology of the male and female genitalia follows Dupuis (1955) and Pericart (1987). The photographs were compared with material from the Naturhistoriska Riksmuseet of Stockholm, Sweden (http://www.nrm.se/2.1286b10fdbe80efba80001.html) and the American Museum of Natural History of New York (http://www.amnh.org/). Descriptions were taken from http://www.biodiversitylibrary.org/ , http://archive.org/ . The diagnosis of the genus *Pagasa*, *Metatropiphorus* and *Nabis* were taken from Blatchley (1926) (http://www.biodiversitylibrary.org/item/29937); and the diagnosis of *Lasiomerus* and *Hoplistoceslis* from Champion (1899) (http://www.biodiversitylibrary.org/item/14631#page/311/mode/1up), Harris (1928) and Reuter (1890) (http://www.archive.org/search.php?query=Revue%20d%C2%B4Entomologie%201890). Specimen locations were georeferenced using Google Earth 6.1.0.4738 (beta) (Google Inc. 2011). Location decimal degree coordinates were processed with DIVA-GIS 7.1.7 (http:// www.diva-gis.org/) to generate species distribution maps.

## Results

### Key to species of Nabidae from Argentina

**Table:** 

1	Scutellum with 1–7 pairs of conspicuous trichobothria along lateral margins (Fig. 1c). Legs short and thick, fore femur usually strongly incrassate; labium relatively short and stout; antennae four segmented, plus one extra at the base of antennal segment II, which is half or more than half of the length of antennal segment I (Fig. 1b). Body shiny black. (Subfamily Prostemmatinae Reuter)	2
–	Scutellum without trichobothria; legs long and thin, fore femur at most only moderately incrassate; rostrum relatively slender and elongate; antennae long with four segments, and sometimes an additional ring-shaped segment at the base of the second; not shiny black. (Subfamily Nabinae Costa)	4
2	Rostral segment II shorter than III, not extending beyond hind margins of eyes (Subgenus *Lampropagasa* Reuter)	*Pagasa fuscipennis* Reuter (Fig. 1a–k)
–	Rostral segment II longer than III, extending distinctly beyond hind margins of eyes (Subgenus *Pagasa* Stål)	3
3	Rostrum reaching from the middle to the apex of fore coxae. Fore trochanter with 4–6 minute black teeth, middle and hind trochanters without teeth. Fore femur strongly thickened	*Pagasa costalis* Reuter
–	Rostrum reaching the middle of the mesothorax. Fore, middle and hind trochanters without teeth. Fore femur moderately thickened	*Pagasa signatipennis* Reuter
4	First antennal segment twice as long as the head and thickened on apical third	*Metatropiphorus alvarengai* Reuter
–	First antennal segment never twice the long of the head and not thickened apically	5
5	Fore and middle legs with rows of long and rigid spines (Fig. 2a)	*Lasiomerus constrictus* Champion (Fig. 3)
–	Fore and middle legs without rows of long and rigid spines	6
6	Fore and middle femora armed beneath with minute, short, rather blunt piceous teeth (Fig. 2b)	*Hoplistoscelis sordida* Reuter (Fig. 4a–c)
–	Fore and middle femora with short dense setae shiny, without teeth (Fig. 2c)	7
7	Body robust and shining, sparsely covered with fine, whitish pubescence	*Nabis tandilensis* Berg (Fig. 5)
–	Body not robust and shining, covered with abundant whitish pubescence	8
8	Body slender and elongate	9
–	Body not slender and elongate	10
9	Hemelytra clear and hyaline. Second segment of rostrum slightly longer than the third. Pronotum slightly longer than broad (Subgenus *Tropiconabis* Kerzhner)	*Nabis capsiformis* Germar (Fig. 6a–j)
–	Hemelytra not clear and hyaline. Second segment of rostrum slightly shorter than the third. Pronotum slightly broader than long	*Nabis setricus* Harris
10	Venter uniformly sordid brown. Hemelytra short in brachypterous form, reaching onto the middle of the first dorsal segment of the abdomen	*Nabis roripes* Stål
–	Venter not sordid brown. Hemelytra in brachypterous form surpassing the middle of the first dorsal segment of the abdomen	11
11	Length of first antennal segment equal to or slightly shorter than the distance between the eyes. Length of second antennal segment subequal to the width of the base of the pronotum. Membrane in brachypterous form slightly surpassing the apex of the corium	*Nabis paranensis* Harris (Fig. 7a–i)
–	Length of first antennal segment markedly shorter than the distance between the eyes. Second antennal segment shorter than the width of the base of the pronotum. Membrane in brachypterous form widely surpassing the apex of the corium	12
12	Pronotum in macropterous form greatly expanded behind, a third wider than long. Pronotum in brachypterous form visibly wider than long, but strongly compressed on the sides between the anterior and posterior lobes	*Nabis argentinus* Meyer-Dür (Fig. 8a–i)
–	Pronotum in macropterous form not more than one fifth wider than long. Pronotum in brachypterous form as wide as long, anterior and posterior lobes not so markedly different	13
13	Body and legs robust. Eyes large. Length of antennal segment II hardly equal to the width of the head across eyes	*Nabis faminei* Stål (Fig. 9a–i)
–	Body and legs more slender. Eyes smaller. Length of antennal segment II slightly longer than width of the head across eyes	*Nabis punctipennis* Blanchard (Fig. 10a–j)

### Subfamily Prostemmatinae Reuter, 1890. Tribe Prostemmatini Reuter, 1890

#### Genus Pagasa

Stål, 1862

##### Diagnosis.

Black or fuscous shining species, having the eyes large, prominent and coarsely granulated; pronotum longer than broad with a fine, straight, transverse groove very close to front margin; scutellum with two small median fovea; embolium of hemelytra present; front and middle tibiae with a spongy fossa at apex.

#### Subgenus Pagasa (Lampropagasa)

Reuter, 1909

##### Diagnosis.

Rostral segment II shorter than or as long as segment III, in most species not surpassing hind margin of eye. Corium and clavus uniformly (strongly or moderately) shining throughout. Both veins of corium (R+M and Cu) or at least the inner vein (Cu) distinct up to hind margin of corium. Vein Cu with punctures (obsolete in some species) on both sides.

#### Pagasa fuscipennis

Reuter, 1909

http://species-id.net/wiki/Pagasa_fuscipennis

Figs 1a–k , 13a

Pagasa nitida Berg 1884. Annales de la Sociedad Científica Argentina 2: 105.
Pagasa fuscipennis Reuter and Poppius 1909. Acta Societatis Scientiarum Fennicae 37: 30. Pennington 1921. Lista de los Hemipteros de la Républica Argentina. Segunda Parte: 26. Harris 1939. Notas del Museo de La Plata 26: 369. Ruffinelli and Pirán 1959. Boletín de la Facultad de Agronomía de Montevideo 51: 41. Kerzhner and Konstantinov 2008. Zoosystematica Rossica 17: 38. Volpi and Coscarón 2010. Zootaxa 2513: 62.

##### Material examined.

ARGENTINA: BUENOS AIRES: Prov. de Buenos Aires, J. Bosq. 1 ♀ (MLP); Ciudad de Buenos Aires 34°36'30.30"S, 58°22'23.38"W, 25–XII–1918 Bosq. col., 1 ♀ (MLP); Ciudad de Buenos Aires, 9–III–1914, 1 ♀ (MLP); Caballito 34°37'0.00"S, 58°27'0.00"W, I–1928 Bosq. col., 1 ♀ (MLP); Chacabuco 34°38'9.36"S, 60°27'54.44"W, F. Lynch col., Harris det. 1 ♀ (MLP). CHACO: Km 42, 18–V–1936 P. Denier det., 1 ♀ (MLP); Resistencia 27°27'5.96"S, 58°59'10.51"W, 21–I–1939 Harris det. 1 ♀ (MLP). SANTIAGO DEL ESTERO: Río Salado, Wagner col., 1 ♀ (MLP). URUGUAY: Montevideo, Colón 34°48'8.30"S, 56°13'26.90"W, 12–I–1929 A. Montora col., Harris det., 1 ♀ (MLP).

##### Distribution in Argentina.

Buenos Aires: Caballito, Ciudad de Buenos Aires, Chacabuco, Ciudad de Bs. As., San Fernando, Tandil; Chaco: Resistencia; Chubut: Rio Turbio, El Maiten, Pedregos Epuyen; Córdoba: Alta Gracia Misiones; Río Negro: Ñorquinco, El Bolsón; Salta: Metan; San Luis; Santa Fé: Ciudad de Santa Fé.

##### Distribution outside Argentina.

Brazil: Goyas, São Paulo, Minas Gerais, Santa Catarina; Paraguay: Asunción, Horqueta; Uruguay: Colonia, Montevideo, Soriano.

##### Measurements.

Female (n = 5): Length 5.00–6.36 (mean = 5.96). Head: length 0.82–0.90 (mean = 0.85), width 0.82–0.91 (mean = 0.89); eye width 0.41–0.48 (mean = 0.45), interocular width 0.29–0.33 (mean = 0.30). Rostrum: ratio of segment lengths about 1: 1.96 : 2.28 : 1. Antenna: ratio of segment about 1: 0.57 : 2.48 : 2.27 : 2.21. Pronotum length 1.35–1.59 (mean = 1.47), width 1.69–1.88 (mean = 1.77). Hemelytra length 2.07–4.05 (mean = 3.47). Abdomen: length 2.17–2.66 (mean = 2.47), width 1.93– 2.18 (mean = 2.07). Legs: fore femora: length 1.24–1.35 (mean = 1.31), width 0.42–0.53 (mean = 0.47); middle femora: length 1.21–1.40 (mean = 1.33), width 0.27–0.33 (mean = 0.30); hind femora 1.78–1.83 (mean = 1.81), width 0.29–0.33 (mean = 0.31). Fore tibiae: length 1.03–1.25 (mean = 1.12), width 0.21–0.29 (mean = 0.27); middle tibiae: length 1.11–1.35 (mean = 1.24), width 0.14–0.19 (mean = 0.15); hind tibiae: length 1.87–2.12 (mean = 2.01), width 0.09–0.17 (mean = 0.13).

**Figure 1.**
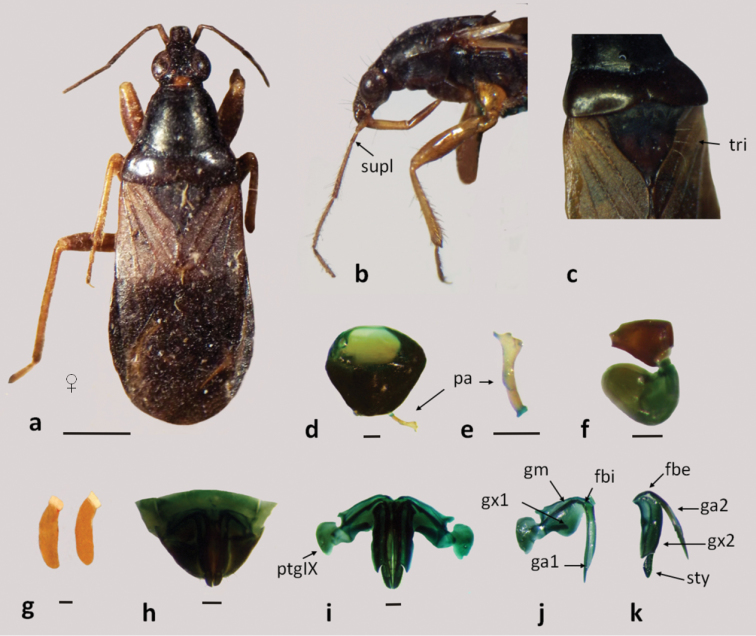
*Pagasa fuscipennis*: **a** dorsal view **b** lateral view **c** scutellum with trichobotrias; **d–f** male genitalia: **d** pygophore **e** paramere **f** aedeagus **g** eggs **h–k** female genitalia: **h–i** genital segment **j** first gonapophysis and gonocoxite 1 **k** second gonapophysis and gonocoxite 2. (fbe: external fibula; fbi: internal fibula, ga1 and ga2: gonapophysis 1 and 2; gm: gonangulum; gxp1 and 2: gonocoxites 1 and 2, pa: paramere, ptgIX: paratergite IX; sty: styloid; supl: supplementary; tri: trichobotria.). Figures **a–c** scale line 1mm; **d–k** scale line 0.2mm.

*Eggs* (Fig. 1g). Length 1.08–1.12 (mean = 1.10; n = 4), width 0.28–0.36 (mean = 0.31; n = 4). Microsculpture absent. Mature eggs were taken from dissected female oviducts.

##### New record.

Argentina: La Pampa, Santa Rosa: 36°37'17.41"S, 64°17'5.91"W, Cornelis col.

#### Subgenus Pagasa (Pagasa)

Stål, 1862

##### Diagnosis.

Rostral segment II longer than or as long as segment III and surpassing hind margin of eye. At least inner half of clavus and inner corner of corium dull, differing from the shining outer part of hemelytron; in most species, the shining part occupies only the outer part of corium outside the medial fracture. Only vein Cu on corium distinct, but obsolete or lacking in hind part of corium. Usually, vein Cu with punctures on outer side only.

#### Pagasa costalis

Reuter, 1909

Pagasa costalis Reuter in Reuter and Poppius 1909. Acta Societatis Scientiarum Fennicae 37: 26–29. Harris 1939. Notas del Museo de La Plata 26: 368. Ruffinelli and Pirán 1959. Boletín de la Facultad de Agronomía de Montevideo, 5l: 40. Kerzhner and Konstantinov 2008. Zoosystematica Rossica, 17: 48.Volpi and Coscarón 2010. Zootaxa, 2513: 63.

##### Distribution in Argentina.

Buenos Aires, Salta.

##### Distribution outside Argentina.

Ecuador: Milagro; Paraguay: Asunción; Surinam: Saramacca; Uruguay: Montevideo.

#### Pagasa signatipennis

Reuter, 1909

http://species-id.net/wiki/Pagasa_signatipennis

Pagasa signatipennis Reuter and Poppius 1909. Acta Societatis Scientiarum Fennicae 37: 26. Harris 1939. Notas del Museo de La Plata 26: 369. Kerzhner and Konstantinov 2008. Zoosystematica Rossica 17: 48. Volpi and Coscarón 2010. Zootaxa 2513: 64.

##### Distribution in Argentina.

Formosa.

##### Distribution outside Argentina.

Bolivia: Rosario, Villa Vicencio; Brazil: Mato Grosso, Santarem; Colombia; Paraguay: Gran Chaco, Horqueta; Surinam: Kwatta, Wagenijen; Venezuela: Sarán de Aquae Apuae.

### Subfamily Nabinae Costa 1853. Tribe Nabini Costa 1853

#### Genus Metatropiphorus

Reuter, 1872

http://species-id.net/wiki/Metatropiphorus

##### Diagnosis.

Elongate, narrow finely pubescent species having the head behind eyes constricted to form a long cylindrical neck; vertex with two fine median grooves; rostrum reaching front coxae; pronotum about as wide at base as long, strongly constricted behind middle, front lobe with a narrow median carina, and side margins distinct; hemelytra surpassing tip of abdomen, membrane large without closed discal cells; fore femora feebly swollen, armed beneath with a number of short distinct spines; fore tibiae setose beneath, apices obliquely truncate.

#### Metatropiphorus alvarengai

Kerzhner, 1987

http://species-id.net/wiki/Metatropiphorus_alvarengai

Fig. 13b

Metatropiphorus alvarengai Kerzhner 1987. Journal New York Entomological Society 95: 569. Holotype (AMNH). Volpi and Coscarón 2010. Zootaxa 2513: 57.

##### Holotype.

http://research.amnh.org/iz/types_db/details.php?specimen_id=5458

##### Distribution in Argentina.

Buenos Aires: Tigre, San Fernando.

##### Distribution outside Argentina.

Brazil: Bahia, Mato Grosso, Santa Catarina. Surinam: Moengo.

#### Genus Lasiomerus

Reuter, 1890

##### Diagnosis.

Posterior lobe of pronotum strongly punctate; hemelytra distinctly constricted before the middle, the costal margin ciliate; femora annulate before the apex; posterior tibiae clothed with long, suberect setae.

#### Lasiomerus constrictus

(Champion, 1899)

http://species-id.net/wiki/Lasiomerus_constrictus

Figs 2a , 3 , 13c

Nabis (Hoplistoscelis) constrictus Champion 1899. Biologia Centrali-Americana 2: 303.
Nabis constrictus Blatchley 1926. Heteroptera or True Bugs of Eastern North America, with especial reference to the faunas of Indiana and Florida: 596.
Nabis (Lasiomerus) constrictus Harris 1928. Entomologica Americana 9: 51.
Lasiomerus constrictus Henry and Lattin 1988. Catalog of the Heteroptera, or True Bugs, of Canada and the Continental United States, p 512. Volpi and Coscarón 2010. Zootaxa 2513: 57.

##### Material examined.

ARGENTINA: BUENOS AIRES: San Isidro 34°28'14.98"S, 58°31'43.00"W, 1 ♀ (NHRS).

##### Distribution in Argentina.

Buenos Aires: San Isidro.

##### Distribution outside Argentina.

Guatemala; Honduras; México: Atoyac, Teapa; Panama: Volcan de Chiriqui. México to Panama.

##### Measurements.

Female (n = 1): Length 6.88. Head: length 0.83, width 0.67; eye width 0.31, interocular width 0.26. Rostrum: ratio of segment lengths about 1: 3.86 : 3.47 : 2.08. Antenna: ratio of segment about 1: 1.56 : 1.56 :1.45. Pronotum length 1.19, width 1.40. Hemelytra length 4.68. Abdomen: length 3.12, width 1.76. Legs: fore femora: length 1.82, width 0.26; middle femora: length 1.71, width 0.20; hind femora 2.34, width 0.15. Fore tibiae: length 1.66, width 0.10; middle tibiae: length 1.71, width 0.10; hind tibiae: length 2.96, width 0.078.

**Figure 2.**
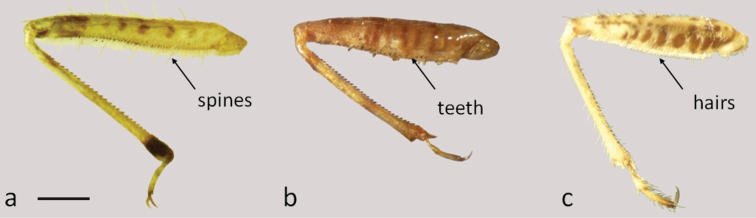
Fore leg: **a** *Lasiomerus constrictus* **b** *Hoplistoscelis sordida* **c** *Nabis argentinus*. Scale line 0.05mm.

**Figure 3.**
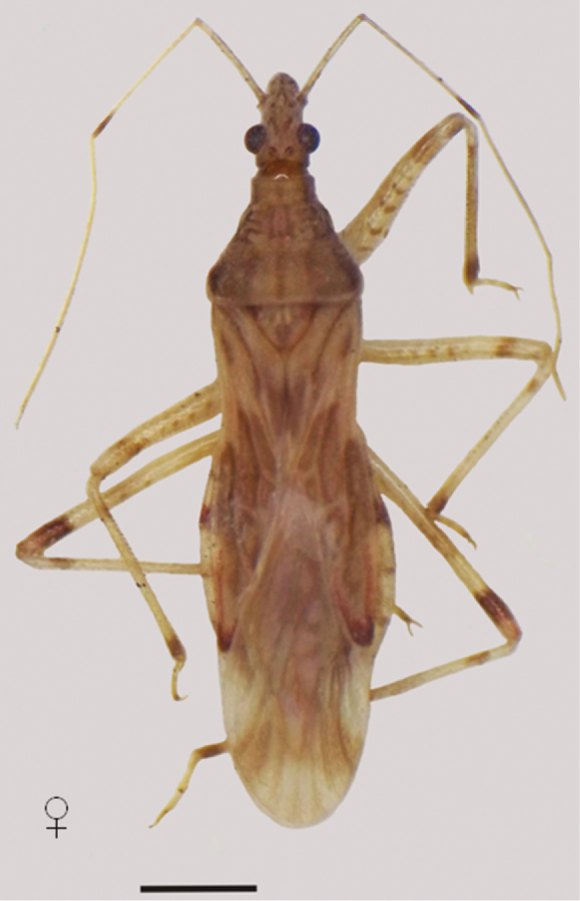
*Lasiomerus constrictus* dorsal view. Scale line 1mm.

##### Description.

Body elongated and light brown. Head covered with abundant long white setae; area between eyes and antennae, and lateral anteocular region brown. Rostrum reaching middle coxae. Antennae long with setae, segment II with a distal band dark brown band; segments III and IV darker than I and II.

##### New records.

1 ♀: Argentina: Buenos Aires: San Isidro 34°28'14.98"S, 58°31'43.00"W, (NHRS).

#### Genus Hoplistoscelis

Reuter, 1890

##### Diagnosis.

Anterior and intermediate femora armed beneath with minute short, rather blunt, piceous teeth; tibiae annulate through out the entire length.

#### Hoplistoscelis sordida

(Reuter, 1872)

http://species-id.net/wiki/Hoplistoscelis_sordida

Figs 2b , 4a–c

Nabis sordidus Reuter 1872. Öfversigt af Kongliga Svenska Vetenskaps-Akademiens Förhandlingar 29: 85. Holotype (NHRS). Champion 1899. Biologia Centrali-Americana 2: 303. Harris 1930. Annals of the Carnegie Museum 19: 241–248.
Hoplistoscelis sordida Kerzhner 1993. Zoosystematica Rossica 1: 39. Volpi and Coscarón 2010. Zootaxa 2513: 56.
Holotype : http://www2.nrm.se/en/het_nrm/s/hoplistoscelis_sordidus.html

##### Material examined.

USA: Iowa: Ames 42°1'38"N, 93°37'54"W, 12–X–1926 H. M. Harris col., Harris det. 1 ♀ (MLP); Davenport 41°33'15"N, 90°36'14"W, 31–VIII–1927 H. G Johnston col., Harris det. 2 ♂♂ (MLP).

##### Distribution outside Argentina.

Brazil. Central and South America from the central part of México to Argentina. Costa Rica: Volcán de Iraza. Eastern North America. Guatemala: Vera Paz, Capetillo. México: Atoyac, Cuernavaca, Guerrero, Orizaba, San Marcos, Teapa, Vera Cruz. Panama: Volcán de Chiriqui. West Indies.

##### Observations.

This species is not mapped because the exact place is not specified, according to Kerzhner (1993) “it occurs in Central and South America from central part of Mexico to Argentina”.

##### Measurements.

Male (n = 2): Length 6.24–6.60 (mean = 6.42). Head: length 0.90–1.01 (mean = 0.95), width 0.82; eye width 0.37, interocular width 0.30–0.33 (mean = 0.31). Rostrum: ratio of segment lengths about 1: 2.45 : 2.63 : 1.36. Antenna: ratio of segment about 1: 1.60: 1.38: 1.34. Pronotum length 1.30, width 1.45–1.60 (mean = 1.52). Hemelytra length 3.91–4.44 (mean = 4.17). Abdomen: length 3.00–3.18 (mean = 3.09), width 1.65– 1.93 (mean = 1.79). Legs: fore femora: length 1.84–1.88 (mean = 1.86), width 0.37); middle femora: length 1.70–1.76 (mean = 1.73), width 0.26; hind femora 2.00–2.25 (mean = 2.12), width 0.18–0.20 (mean = 0.19). Fore tibiae: length 1.54, width 0.11; middle tibiae: length 1.61–1.70 (mean = 1.65), width 0.075; hind tibiae: length 2.44–2.60 (mean = 2.52), width 0.075.

Female(n = 1): Length 6.06. Head: length 0.82, width 0.86; eye width 0.41, interocular width 0.35. Rostrum: ratio of segment lengths about 1: 2 : 3.03 : 4.39. Antenna: ratio of segment about 1: 1.40 : 1.22 : 1.23. Pronotum length 1.27, width 1.20. Hemelytra length 1.12. Abdomen: length 2.70, width 2.25. Legs: fore femora: length 1.88, width 0.41; middle femora: length 1.76, width 0.26; hind femora 2.30, width 0.22. Fore tibiae: length 1.57, width 0.13; middle tibiae: length 1.70, width 0.11; hind tibiae missing.

**Figure 4.**
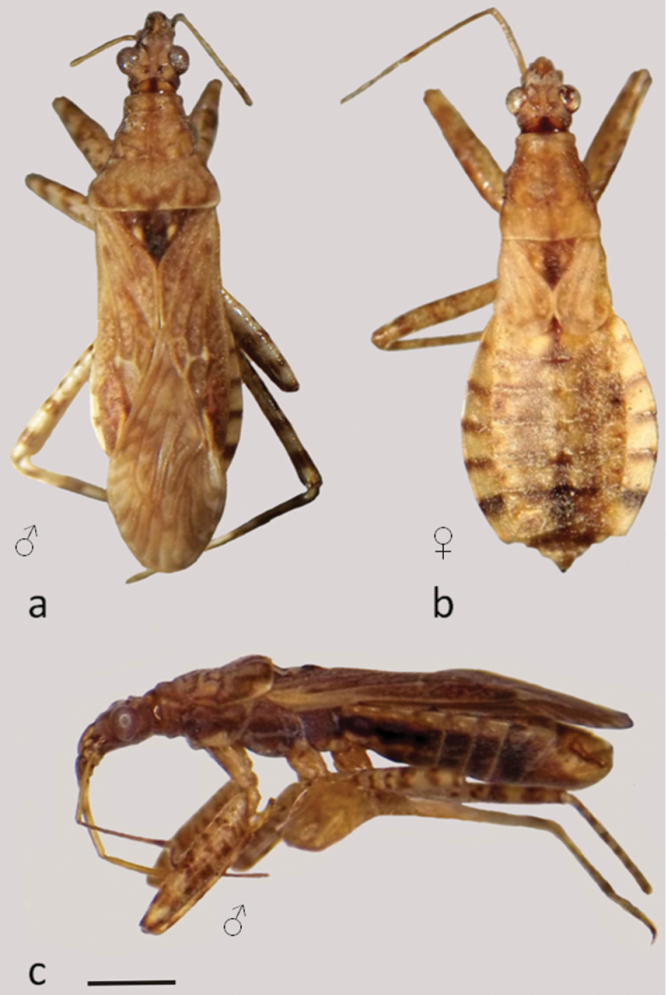
*Hoplistoscelis sordida* **a–b** dorsal view **c** lateral view. Scale line 1mm.

#### Genus Nabis

Latreille, 1802

http://species-id.net/wiki/Nabis

##### Diagnosis.

Body elongate or oblong-oval, usually slender. Pronotum campanulate with two fine transverse constrictions, humeral angles rounded, hind margin subtruncate; scutellum small, triangular, apex obtuse; clavus widened behind; hemelytra entire reaching or surpassing tip of abdomen, or abbreviated reaching only second dorsal segment, membrane with three elongate cells, their terminal bounding vein with numerous short veins radiating to tip of membrane; connexivum in males of macropterous forms usually narrowly or not at all exposed, in females more broadly so; front femora spindleshaped, moderately swollen, femora and tibiae beset beneath with numerous fine setae; front and middle tibiae with a short spongy lobe at apex; tarsi 3-jointed.

#### Nabis tandilensis

(Berg, 1884)

http://species-id.net/wiki/Nabis_tandilensis

Figs 5 , 13d

Coriscus tandilensis Berg 1884. Annales de la Sociedad Científica Argentina 2: 106.
Nabis tandilensis Pennington 1921. Lista de los Hemipteros de la República Argentina. Segunda Parte: 26. Harris 1939. Notas del Museo de La Plata 26: 377. Holotype (MLP). Volpi and Coscarón 2010. Zootaxa 2513: 59.*Holotype*: ♂, Argentina, Buenos Aires: Tandil (MLP)

##### Material examined.

Holotype ♂ BUENOS AIRES: Tandil 37°19'4.12"S, 59°9'1.41"W, 5–XI–1983 Doctor Holmberg, (MLP).

**Figure 5.**
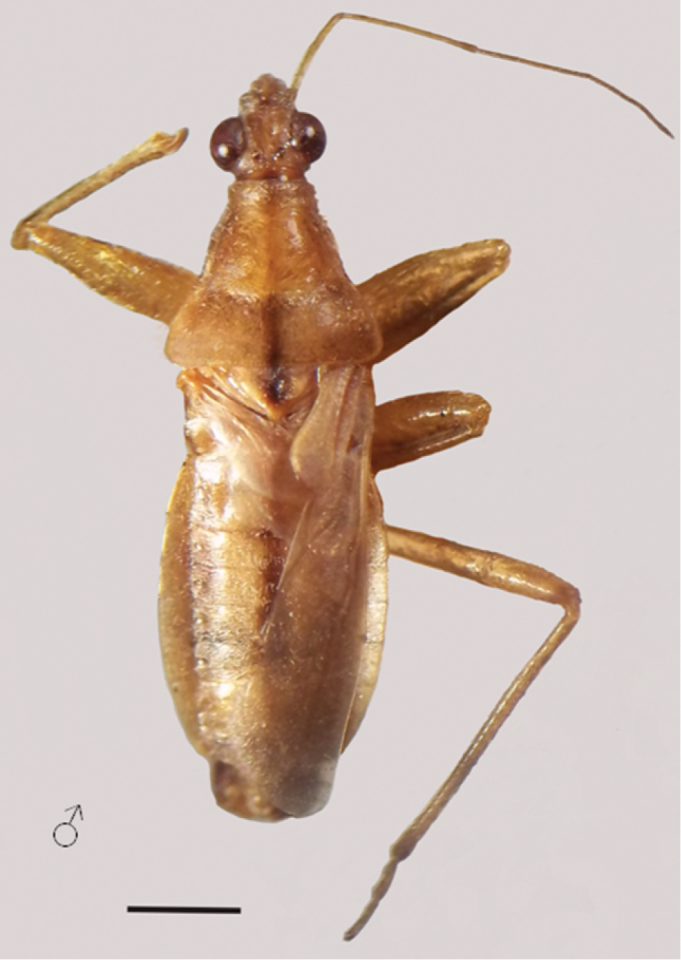
*Nabis tandilensis* dorsal view. Scale line 1mm.

##### Distribution in Argentina.

Buenos Aires: Tandil.

#### Genus Tropiconabis

Kerzhner, 1968

##### Diagnosis.

Body long and narrow, antennae and legs long, hemelytra surpassing tip of abdomen. Abdomen usually with clear edges. Paramere small.

#### Nabis capsiformis

Germar, 1837

http://species-id.net/wiki/Nabis_capsiformis

Figs 6a–j , 13d

Nabis capsiformis Germar 1837. Silbermann’s Revue Entomologique 5: 132. Pennington 1920–1921. Lista de los Hemípteros Heterópteros de la República Argentina. Segunda Parte: 26. Harris 1930. Annals of the Carnegie Museum Argentina 19: 246. Harris 1939. Notas del Museo de La Plata 26: 376. Ruffinelli and Pirán 1959. Boletín de la Facultad de Agronomía de Montevideo 5l: 40. Prado 2008. Boletín del Museo Nacional de Historia Natural Chile 57: 44. Volpi and Coscarón 2010. Zootaxa 2513: 60.
Nabis elongatus Meyer-Dür 1870. Mitteilungen der Schweizerischen Entomologischen Gesellschaft 3:178. Synonymized by Reuter 1908: 114.
Nabis kinbergii Reuter 1872. Öfversigt af Kongliga Svenska Vetenskaps-Akademiens Forhandlingar 29: 90. Synonymized by Reuter 1908: 114.
Coriscus capsiformis Stål 1873. Kongliga Svenska Vetenskaps-Akademiens Handlingar 11: 113.
Coriscus elongatus Stål 1873. Kongliga Svenska Vetenskaps-Akademiens Handlingar 11: 114.
Coriscus kinbergii Stål 1873. Kongliga Svenska Vetenskaps-Akademiens Handlingar 11: 113.
Reduviolus capsiformis Reuter 1908 Mémoires de la Société Entomologique de Belgique 15: 114.

##### Material examined.

BUENOS AIRES: Bs. As. 1 ♀ (MLP); J. Bosq col., 1 ♀ (MLP); 1776 Harris det., 1 ♂ (MLP); Alm. Brown 34°50'24.22"S, 58°23'40.24"W, 19–V–36 1 ♀ (MLP); Arrecifes 34°3'49.96"S, 60°6'12.56"W, 17–I–1939 Biraben-Scott leg. col., 1 ♀ (MLP); Ciudad de Buenos Aires 34°36'30.30"S, 58°22'23.38"W, XI–1918 1 ♀ (MLP), 12–II–1912 1 ♀ (MLP); José C. Paz 34°30'54.38"S, 58°45'58.49"W, XI–1958 1 ♀ (MLP), XII–1911 1 ♂ (MLP), 1940 J. A. Rosas Costa leg. col., 1 ♂, 2 ♀♀ (MLP); La Plata 34°55'2.28"S, 57°57'0.47"W, 1 ♀ (MLP), Harris det., 1 ♂, 1 ♀ (MLP); Luján 34°33'56.63"S, 59°7'2.76"W, 18–XII–58 2 ♂♂, 3 ♀♀ (MLP); Mar del Plata 37°58'47.49"S, 57°35'23.26"W, 5–XII–1938 Biraben-Scott leg. col., 1 ♂ (MLP); Punta de Indio 35°16'27.66"S, 57°15'38.66"W, 4–XII–1938 Biraben-Scott leg. col., 2 ♀♀ (MLP); Rincón de Noario 8–IX–1935, 1 ♀ (MLP); San Nicolas 34°36'19.00"S, 58°22'33.00"W, Biraben-Scott leg. col., 1 ♀ (MLP). CATAMARCA: Pomán 28°23'47.29"S, 66°13'7.56"W, 8–III–62 Torres-Ferreyra col., 1 ♀ (MLP). CORDOBA: Alta Gracia 31°39'16.39"S, 64°25'50.17"W, I–35 C. Bruch leg. col., 1 ♀ (MLP); Cabana 31°13'0.01"S, 64°22'0.01"W, 03–I–1926 Harris det., 1 ♀ (MLP), 10–XI–1942 Biraben col., 1 ♂ (MLP), Marull 30°59'45.16"S, 62°49'37.61"W, 22–I–1940 Biraben, 1 ♂ (MLP); San Antonio de Arredondo 31°28'57.22"S, 64°31'25.50"W, 14–II–1940 Biraben col., 1 ♂, 1 ♀ (MLP). CORRIENTES: Harris det., 1 specimen (without abdomen) (MLP); I–1921 De Carlo col., 1 ♂ (MLP); San Roque 28°34'28.86"S, 58°42'32.85"W, II–1920 1 ♂, 2 ♀♀ (MLP). JUJUY: Pampa Blanca 24°31'58.57"S, 65°4'24.57"W, 13–III–1939 Biraben-Scott leg. col., 1 ♂ (MLP). MISIONES: Loreto 27°19'0.01"S, 55°31'59.98"W A. A. Orgloblin col., 2 ♀♀ (MLP). SANTIAGO DEL ESTERO: Girardet 27°37'0.02"S, 62°10'0.02"W, 9–XII–1939 Biraben–Bezzi, 2 ♀♀ (MLP); Quimilí 27°38'39.06"S, 62°24'56.03"W, 9–XII–1939 Biraben–Bezzi col., 1 ♀ (MLP).

##### Distribution in Argentina.

Buenos Aires: Alm. Brown, Arrecifes, Ciudad de Buenos Aires, José C. Paz, La Plata, Luján, Mar del Plata, Punta de Indio, Rincón de Noario, San Nicolas; Catamarca: Pomán; Córdoba: Alta Gracia, Cabana, Marull, San Antonio de Arredondo; Corrientes: San Roque; Jujuy: Pampa Blanca; La Pampa: Winifreda; Misiones: Loreto, Río Bermejo, Salto; Salta (Río Bermejo); Santiago del Estero: Girardet, Quimilí.

##### Distribution outside Argentina.

Brazil: Santaren, Corumbá, Rio de Janeiro; Chile: Arica, Continental Chile and Easter Island; Guyana; México; Peru: Lima; Uruguay: Montevideo.

According to Kerzhner (2007) this species is widely distributed in nearly all tropical and subtropical regions of the world, the Americas from the USA to Argentina.

##### Measurements.

Male(n = 5): Length 8.54–9.10 (mean = 8.74). Head: length 0.92–1.06 (mean = 1.00), width 0.71–0.72 (mean = 0.712); eye width 0.28–0.33 (mean = 0.31), interocular width 0.28–0.32 (mean = 0.30). Rostrum: ratio of segment lengths about 1: 2.59 : 2.73 : 1.38. Antenna: ratio of segment about 1: 1.75 : 1.76 : 1.07. Pronotum length 1.19–1.35 (mean = 1.25), width 1.27–1.42 (mean = 1.36). Hemelytra length 6.10–6.60 (mean = 6.38). Abdomen: length 3.05–3.76 (mean = 3.38), width 1.14–1.70 (mean = 1.45). Legs: fore femora: length 2.13–2.16 (mean = 2.14), width 0.31–0.36 (mean = 0.34); middle femora: length 1.87–1.98 (mean = 1.95), width 0.12–0.23 (mean = 0.16); hind femora 3.05–3.26 (mean = 3.13), width 0.12–0.15 (mean = 0.13). Fore tibiae: length 1.70–1.86 (mean = 1.79), width 0.10; middle tibiae: length 1.77–1.92 (mean = 1.84), width 0.05–0.07 (mean = 0.064); hind tibiae: length 3.48–3.76 (mean = 3.66), width 0.05–0.07 (mean = 0.064).

Female(n = 5): Length 7.77–9.87 (mean = 8.85). Head: length 0.98–1.13 (mean = 1.03), width 0.71–0.78 (mean = 0.73); eye width 0.31–0.35 (mean = 0.33), interocular width 0.28–0.32 (mean = 0.30). Rostrum: ratio of segment lengths about 1: 2.42 : 2.57 : 1.28. Antenna: ratio of segment about 1: 1.69 : 1.73 : 1.07. Pronotum length 1.27–1.42 (mean = 1.34), width 1.42–1.63 (mean = 1.51). Hemelytra length 5.53–7.17 (mean = 6.44). Abdomen: length 3.12–3.69 (mean = 3.46), width 1.04–1.56 (mean = 1.32). Legs: fore femora: length 2.08–2.27 (mean = 2.23), width 0.35–0.39 (mean = 0.37); middle femora: length 1.91–2.13 (mean = 2.03), width 0.20–0.28 (mean = 0.22); hind femora 3.12–3.33 (mean = 3.2), width 0.12–0.26 (mean = 0.19). Fore tibiae: length 1.82–1.91 (mean = 1.86), width 0.10–0.12 (mean = 0.104); middle tibiae: length 1.91–2.05 (mean = 1.97), width 0.07–0.12 (mean = 0.08); hind tibiae: length 3.74–3.97 (mean = 3.85), width 0.07.

##### Description.

General coloration light brown. Body elongated, covered with white setae over the surface. Head with whitish pilosity and sparse long setae, more abundant ventrally; brown area between eyes and antennae and post-ocular region laterally. Rostrum passing fore coxae, segment IV brown distally. Antennae long and slender with setae. Pronotum pilose with a brown stripe in the middle (in some specimens diffused); anterior lobe tinged with brown; posterior lobe with a suture and granulate. Scutellum brown in the centre and with two depressions, and the sides clear. In some specimens meso- and metasternum dark brown. Pro- meso- and metapleura and abdomen ventral sides with a brown stripe. Abdomen with abundant sparse setae, not uniformly pigmented, connexivum without spots. Legs long and slender, with long white setae.

**Figure 6.**
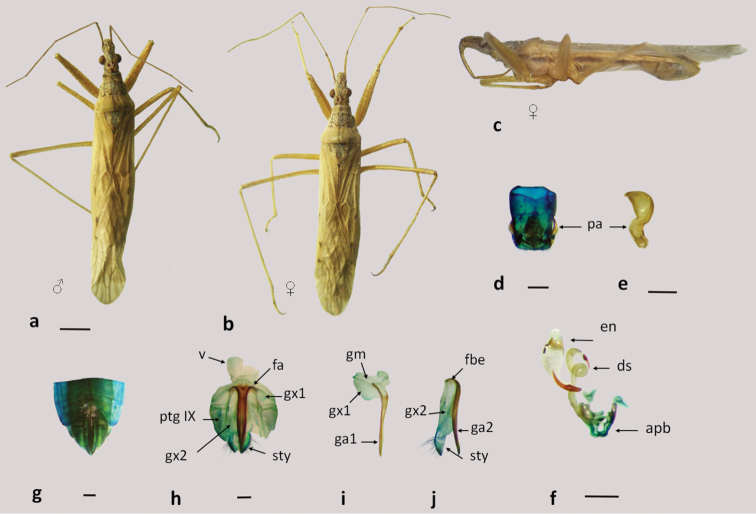
*Nabis capsiformis* **a–b** dorsal view **c** lateral view; **d–f** male genitalia: **d** pygophore **e** paramere **f** aedeagus **g–j** female genitalia: **g–h** genital segment **i** first gonapophysis and gonocoxite 1 **j** second gonapophysis and gonocoxite 2. (apb: articulatory apparatus; ds: ductus seminis; en: endosoma; fa: anterior fibula; fbe: external fibula; ga1 and ga2: gonapophysis 1 and 2; gm: gonangulum; gx1 and 2: gonocoxites 1 and 2, pa: paramere, ptgIX: paratergite IX ; sty: styloid; v: vagina). Figures **a–c** scale line 1mm; **d–j** scale line 0.2mm.

##### Biology.

Ojeda-Peña (1971) and Cornelis et al. (2012) described the nymphs, eggs, and biology. The last authors collected the material using a sweeping net in *Medicago sativa* L. (Fabaceae).

#### Nabis seticrus

Harris, 1930

Nabis seticrus Harris 1930. Annals of the Carnegie Museum, 19:241–248. Volpi and Coscarón 2010. Zootaxa 2513: 59.

##### Distribution in Argentina.

Salta

##### Distribution outside Argentina.

Brazil: Chapada, Rio de Janeiro.

#### Nabis roripes

Stål, 1860

http://species-id.net/wiki/Nabis_roripes

Nabis roripes Stål 1860. Kongliga Svenska Vetenskaps-Akademiens Handlingar 2: 70. Holotype (NHRS). Reuter 1890. Revue d´Entomologie 9: 297. Reuter 1908. Mémoires de la Société Entomologique de Belgique 15: 99–101. Harris 1930. Annals of the Carnegie Museum 19: 246. Harris 1939. Notas del Museo de La Plata 26: 376. Volpi and Coscarón 2010. Zootaxa 2513: 59.

##### Distribution in Argentina.

*Holotype*: http://www2.nrm.se/en/het_nrm/r/nabis_roripes.html

Misiones: Loreto.

##### Distribution outside Argentina.

Brazil: Chapada; Colombia: Pandi (Cundimarca); Peru: San Juan.

### *Punctipennis* complex

The *punctipennis* complex (according to Harris (1939)) is comprised of *Nabis argentinus* Meyer-Dür, *Nabis faminei* Stål, *Nabis paranensis* Harris, and *Nabis punctipennis* Blanchard.

Body with abundant long whitish setae. Head brown; with a line in the middle (widened anteriorly), sides of head and ventrally dark brown. Rostrum pallid testaceus, segment I darker at base, IV distally darker. Pronotum with a dark brown stripe in the middle, anterior lobe with irregular fuscous patterns. Scutellum black with a yellowish spot at the sides of the base; with two fovea in the center the area between noticeably depressed. Abdomen above and a wide line on each side sordid brown. Legs pilose, mottled.

#### Nabis paranensis

Harris, 1931

http://species-id.net/wiki/Nabis_paranensis

**Figure 7.**
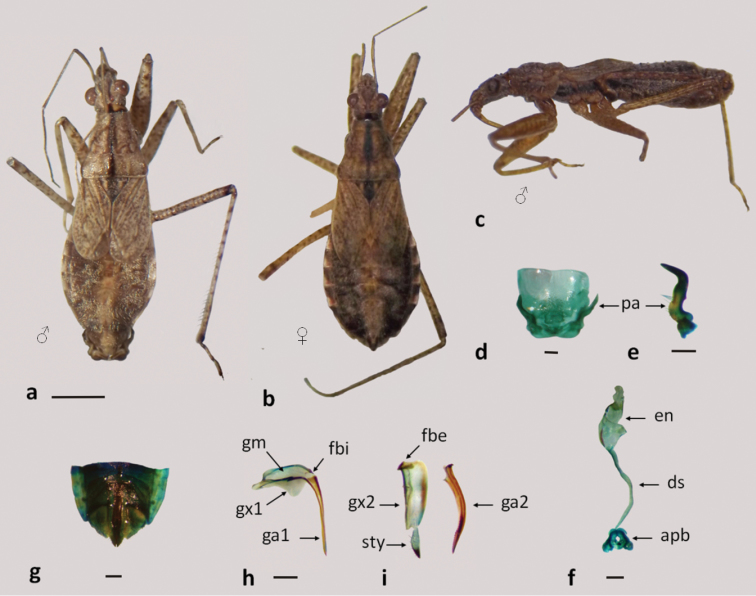
*Nabis paranensis* **a–b** dorsal view **c** lateral view **d–f** male genitalia: **d** pygophore **e** paramere **f** aedeagus **g–i** female genitalia: **g** genital segment **h** first gonapophysis and gonocoxite 1 **i** second gonapophysis and gonocoxite 2. (apb: articulatory apparatus; ds: ductus seminis; en: endosoma; fbe: external fibula; fbi: internal fibula; ga1 and ga2: gonapophysis 1 and 2; gm: gonangulum; gx1 and 2: gonocoxites 1 and 2, pa: paramere; sty: styloid). Figures **a–c** scale line 1mm; **d–i** scale line 0.2mm.

Figs 7a–i , 11a , 12a , 13d

Nabis paranensis Harris 1931. Annals of the Zoological Museum of Polonian 9: 182. Harris 1939. Notas del Museo de La Plata 26: 374. Volpi and Coscarón 2010. Zootaxa 2513: 58.

##### Material examined.

BUENOS AIRES: 1 ♂ (MLP); José C. Paz 34°30'54.38"S, 58°45'58.49"W, 1940 J. A. Rosas Costa leg. Col., Harris det., 1 ♂ (MLP); La Plata 34°55'2.28"S, 57°57'0.47"W A. R. Bezzi leg. col., 1 ♀ (MLP); V. Ballester 34°32'57.25"S, 58°33'31.75"W, 12–VII–1938 1 ♂ (MLP); V. Devoto 34°36'0.00"S, 58°30'60.00"W, 05–III–1939 1 ♂ (MLP). JUJUY: Termas de Reyes 24°10'16.60"S, 65°29'10.62"W, 27–XII–1971 L. Herman col., Kerzhner det. 1985, 1 ♂ (AMNH); Yala 24°7'10.77"S, 65°24'14.34"W, 12–III–1939 Biraben-Scott leg. col., 1 ♂, 2 ♀♀ (MLP). LA PAMPA: E. Castex 35°54'49.15"S, 64°17'19.94"W, 31–I–1957 Torres-Ronderos col., 2 ♀♀ (MLP); Parque Luro 36°57'34.32"S, 64°15'7.15"W, 26–I–1957 Torres-Ronderos col., 1 ♀ (MLP). MISIONES: Delta Paraná Guazú 29–II–1919, Bosq col., 1 ♂ (MLP).

##### Distribution in Argentina.

Buenos Aires: José C. Paz, La Plata, V. Ballester, V. Devoto; Jujuy: Termas de Reyes, Yala; La Pampa: Eduardo Castex, Parque Luro; Misiones: Delta Paraná Guazú.

##### Distribution outside Argentina.

Brazil: Parana (São Pedro de Mallet).

##### New record.

Argentina: La Pampa, Winifreda: 36°18'45.30"S, 64°11'55.45"W, Cornelis col.

##### Measurements.

Male(n = 5): Length 5.17–6.30 (mean = 5.53). Head: length 0.79–1.00 (mean = 0.89), width 0.79–0.85 (mean = 0.81); eye width 0.32–0.42 (mean = 0.35), interocular width 0.33–0.35 (mean = 0.33). Rostrum: ratio of segment lengths about 1: 2.28 : 1.97 : 1.11. Antenna: ratio of segment about 1: 1.59 : 1.32 : 1.09. Pronotum length 1.09–1.24 (mean = 1.14), width 1.12–1.40 (mean = 1.22). Hemelytra length 1.16–3.94 (mean = 1.91). Abdomen: length 2.21–3.50 (mean = 2.83), width 1.46–2.48 (mean = 1.71). Legs: fore femora: length 1.61–1.98 (mean = 1.74), width 0.35–0.41 (mean = 0.38); middle femora: length 1.42–1.63 (mean = 1.53), width 0.26–0.30 (mean = 0.27); hind femora 2.06–2.48 (mean = 2.22), width 0.14–0.18 (mean = 0.17). Fore tibiae: length 1.31–1.61 (mean = 1.41), width 0.10–0.11 (mean = 0.108); middle tibiae: length 1.35–1.65 (mean = 1.45), width 0.07–0.11 (mean = 0.08); hind tibiae: length 2.33–2.98 (mean = 2.57), width 0.07–010 (mean = 0.076).

Female(n = 5): Length 5.45–5.89 (mean = 5.67). Head: length 0.75–1.00 (mean = 0.88), width 0.82–0.86 (mean = 0.84); eye width 0.35–0.39 (mean = 0.37), interocular width 0.33–0.37 (mean = 0.35). Rostrum: ratio of segment lengths about 1: 2.23 : 2.41 : 1.17. Antenna: ratio of segment about 1: 1.51 : 1.30 : 1.03. Pronotum length 1.12–1.24 (mean = 1.17), width 1.20–1.35 (mean = 1.28). Hemelytra length 1.50–2.10 (mean = 1.79). Abdomen: length 2.70–3.00 (mean = 2.84), width 1.69–1.95 (mean = 1.84). Legs: fore femora: length 1.61–2.06 (mean = 1.87), width 0.33–0.43 (mean = 0.39); middle femora: length 1.70–2.05 (mean = 1.84), width 0.22–0.26 (mean = 0.24); hind femora 1.80–2.63 (mean = 2.26), width 0.15–0.18 (mean = 0.17). Fore tibiae: length 1.27–1.61 (mean = 1.49), width 0.09–0.11 (mean = 0.10); middle tibiae: length 1.39–1.80 (mean = 1.60), width 0.07–0.11 (mean = 0.08); hind tibiae: length 2.63–3.15 (mean = 2.78), width 0.07.

##### Description.

Eyes prominent; antennae and legs longer than in the others species of the complex. Length of first antennal segment equal or slightly shorter than the distance between the eyes. Length of second antennal segment subequal to the width of the base of the pronotum. In brachypterous forms, hemelytra reaching to the fifth abdominal segment and membrane slightly surpassing the apex of the corium. Paramere distally shorter and more thickened than in *Nabis faminei* and *Nabis punctipennis*. Paramere with a thickening on the inner margin of the blade and a protuberance on the outer margin of the blade. Female genitalia with the styloid constricted basally and sharp distally.

#### Nabis argentinus

Meyer-Dür, 1870

http://species-id.net/wiki/Nabis_argentinus

**Figure 8.**
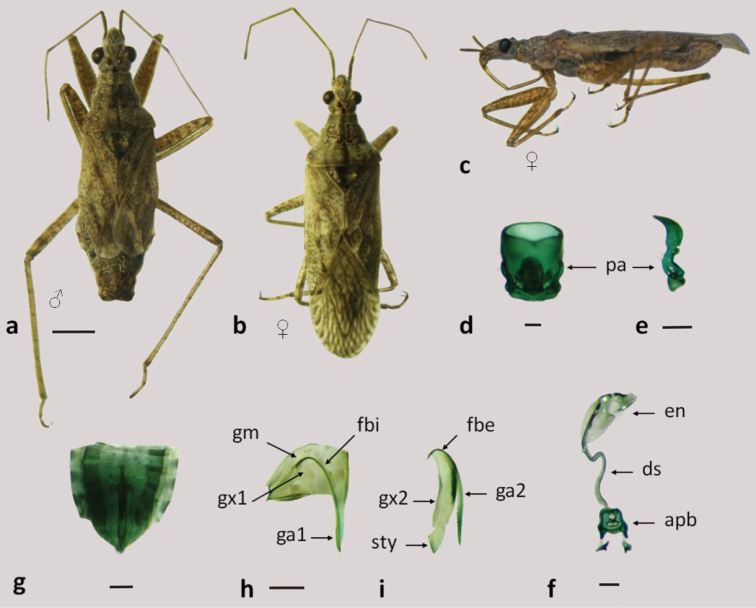
*Nabis argentinus* **a–b** dorsal view **c** lateral view **d–f** male genitalia: **d** pygophore **e** paramere **f** aedeagus **g–i** female genitalia: **g** genital segment **h** first gonapophysis and gonocoxite 1 **i** second gonapophysis and gonocoxite 2. (apb: articulatory apparatus; ds: ductus seminis; en: endosoma; fbe: external fibula; fbi: internal fibula; ga1 and ga2: gonapophysis 1 and 2; gm: gonangulum; gx1 and 2: gonocoxites 1 and 2, pa: paramere; sty: styloid). Figures **a–c** scale line 1mm; **d–i** scale line 0.2mm.

Figures 8a–i , 11b , 12b , 13d

Nabis argentinus Meyer-Dür 1870. Mitteilungen der Schweizerischen Entomologischen Gesellschaft 3: 177. Harris 1939. Notas del Museo de La Plata 26: 373. Ruffinelli and Pirán 1959. Boletín de la Facultad de Agronomía de Montevideo 5l: 40. Volpi and Coscarón 2010. Zootaxa 2513: 58.
Coriscus argentinus Stål 1873. Köngliga Svenska Vetenskaps-Akademiens Handlingar 11: 114.
Nabis punctipennis :Berg 1879. Anales de la Sociedad Científica Argentina 9: 143. Berg 1892. Anales de la Sociedad Científica Argentina 34: 104. Pennington 1920. Lista de los Hemípteros Heterópteros de la República Argentina. Segunda Parte: 26.

##### Material examined.

BUENOS AIRES: 1852 Harris det., 1 ♀ (MLP), 15–I–1921 1 ♂ (MLP), XII–1938 Drake and Richardson col., Harris det., 1 ♂, 1 ♀ (MLP); Bahía Blanca 38°42'42.04"S, 62°16'5.08"W, III–1984 Mianzan col., 3 ♀♀ (MLP), 18–II–1977 S. Coscarón col., Kerzhner det. 1987, 3 ♂♂, 3 ♀♀ (AMNH); Baradero 33°48'31.95"S, 59°30'16.86"W, 1 ♂ (MLP), Harris det. 1 ♂ (MLP); Ciudad de Buenos Aires 34°36'30.30"S, 58°22'23.38"W, 24–XII–1918 Bosq col., 1 ♀ (MLP), 25–XII–1918 1 ♀ (MLP), 29–I–1919 Bosq col., 1 ♂ (MLP), 29–III–1919 Bosq col., 1 ♀ (MLP); Flores 34°37'60.00"S, 58°28'0.00"W, 7–III–1917 Bosq col., 1 ♀ (MLP); José C. Paz 34°30'54.38"S, 58°45'58.49"W, XII–1938 1 ♂ (MLP), 07–III–1939 1 ♀ (MLP), 21–I–1939 1 ♂ (MLP), 05–IX–1939, 1 ♀ (MLP), 1940 J. A. Rosas Costa leg. col.; La Colina 37°21'41.73"S, 61°32'2.76"W, 29–XI–1938 Carl J. Drake col., 3 ♂♂, 2 ♀♀ (MLP), 29–XI–1938 Carl J. Drake col., Harris det. 1 ♀ (MLP); La Madrid 37°14'51.31"S, 61°15'38.11"W, 19–XI–1938, 1 ♂ (MLP); La Plata 34°55'2.28"S, 57°57'0.47"W, 1935 J. A. Rosas Costa col., Harris det., 1 ♀ (MLP); Harris det., 1 ♂, 1 ♀ (MLP), A. R. Bezzi leg. col., 1 ♀ (MLP), 03–III–2003 P. M. Dellape col., 1 ♀ (MLP); Monte Hermoso 38°58'58.01"S, 61°17'50.69"W, 28–II–1957 Torres-Ronderos col., 1 ♀ (MLP); Pedro Luro 39°30'5.86"S, 62°41'0.10"W, 08–II–1941 Biraben col., 3 ♀♀ (MLP); Punta del Indio 35°16'27.66"S, 57°15'38.66"W, 4–XII–1938 Biraben-Scott leg. col., 1 ♂ (MLP); Tandil 37°19'14.28"S, 59°7'44.78"W, Harris det. 1 ♀ (MLP). CATAMARCA: Belén 27°38'59.42"S, 67°1'59.09"W, 02–III–1939 Biraben-Scott leg col. 2 ♀♀ (MLP). CÓRDOBA: Alta Gracia 31°39'16.39"S, 64°25'50.17"W, I–1935 C. Bruch leg. Col., Harris det., 1 ♀ (MLP); Bajo Grande 31°37'0.02"S, 64°13'0.00"W, 07–XII–1939 Biraben-Bezzi col., 1 ♀ (MLP); Cabana 31°13'0.01"S, 64°22'0.01"W, IX–1938 Biraben-Scott leg. col., 1 ♀ (MLP), 10–II–1942 Biraben col., 1 ♀ (MLP), 10–XI–1942 Biraben col., 1 ♂ (MLP); Copina 31°34'23.82"S, 64°40'25.01"W, 14–II–1940 Biraben col., 1 ♀ (MLP); La Puerta 30°53'38.28"S, 63°15'7.24"W, 23–I–1940 Biraben col., 1 ♀ (MLP); Mar Chiquita 30°47'60.00"S, 62°52'59.99"W, 22–I–1940 Biraben col., 1 ♀ (MLP); Marull 30°59'45.16"S, 62°49'37.61"W, 22–I–1940 Biraben col., 2 ♀♀ (MLP); San Antonio de Arredondo 31°28'57.22"S, 64°31'25.50"W, 14–II–1940 Biraben col., 2 ♀♀ (MLP); San Francisco 31°25'30.00"S, 62°5'2.98"W, 21–I–1940 Biraben col., 1 ♀ (MLP). CHACO: Nueva Pompeya 24°55'53.10"S, 61°28'59.70"W, Harris det., 1 ♀ (MLP). ENTRE RÍOS: Gualeguaychú 33°0'54.06"S, 58°31'9.28"W, 20–XII–1941 Biraben–Bezzi col. 1 ♂ (MLP). LA PAMPA: Eduardo Castex 35°54'49.15"S, 64°17'19.94"W, 31–I–1957 Torres-Ronderos col., 1 ♀ (MLP). MENDOZA: Luján 33°2'1.96"S, 68°52'56.50"W, 27–II–1940 Biraben col., 1 ♂ (MLP); Tupungato 33°22'9.58"S, 69°8'43.59"W, 27–II–1940 Biraben col., 1 ♀ (MLP). RÍO NEGRO: I–1951 1 ♀ (MLP); San Antonio Oeste 40°43'49.84"S, 64°56'57.03"W, 20–II–1915, 2 ♀♀ (MLP). SALTA: San Lorenzo 24°43'46.97"S, 65°29'7.42"W, 14–VII–1939 1 ♀ (MLP). SAN LUIS: Nogolí 32°55'6.09"S, 66°19'30.79"W, 21–II–1940 Biraben col., 1 ♂, 1 ♀ (MLP); Quines 32°14'1.21"S, 65°48'8.50"W, 18–II–1940 Biraben col., 1 ♀ (MLP); San Francisco 33°16'51.13"S, 66°18'32.36"W, 20–II–1940 Biraben col., 1 ♀ (MLP).

##### Distribution in Argentina.

Buenos Aires: Bahía Blanca, Baradero, Ciudad de Buenos Aires, Flores, José C. Paz, La Colina, La Madrid, La Plata, Monte Hermoso, Pedro Luro, Punta del Indio; Catamarca: Belén; Córdoba: Alta Gracia, Bajo Grande, Cabana, Copina, La Puerta, Mar Chiquita, Marull, San Antonio de Arredondo, San Francisco; Chaco: Nueva Pompeya; Entre Ríos: Gualeguaychú; La Pampa: Eduardo Castex; Mendoza: Luján, Tupungato; Río Negro: San Antonio Oeste; Salta: San Lorenzo; San Luis: Nogolí, Quines, San Francisco.

##### Distribution outside Argentina.

Uruguay: Artigas, Canelones, Colonia, Durazno, Maldonado, Montevideo, Paysandú.

##### New record.

Argentina: La Pampa, Winifreda: 36°18'45.30"S, 64°11'55.45"W, Cornelis col.

##### Measurements.

Male (n = 5): Length 5.32–6.81 (mean = 6.34). Head: length 0.78–0.99 (mean = 0.86), width 0.85–0.92 (mean = 0.86); eye width 0.38–0.42 (mean = 0.40), interocular width 0.38–0.42 (mean = 0.41). Rostrum: ratio of segment lengths about 1: 2.54 : 2.48 : 1.29. Antenna: ratio of segment about 1: 1.73 : 1.33 : 0.90. Pronotum length 1.06–1.27 (mean = 1.17), width 1.42–1.77 (mean = 1.57). Hemelytra length 2.48–4.68 (mean = 4.11). Abdomen: length 1.91–2.69 (mean = 2.28), width 1.42–1.77 (mean = 1.65). Legs: fore femora: length 1.70–1.77 (mean = 1.73), width 0.35–0.42 (mean = 0.37); middle femora: length 1.42–2.05 (mean = 1.67), width 0.28–0.32 (mean = 0.29); hind femora 2.27–2.34 (mean = 2.29), width 0.14–0.21 (mean = 0.18). Fore tibiae: length 1.42–1.49 (mean = 1.43), width 0.10–0.12 (mean = 0.105); middle tibiae: length 1.49–1.60 (mean = 1.52), width 0.07–0.10 (mean = 0.09); hind tibiae: length 2.55–2.84 (mean = 2.70), width 0.07–010 (mean = 0.08).

Female(n = 5): Length 5.75–7.10 (mean = 6.57). Head: length 0.88–1.06 (mean = 0.98), width 0.74–0.92 (mean = 0.88); eye width 0.38–0.42 (mean = 0.41), interocular width 0.35–0.42 (mean = 0.39). Rostrum: ratio of segment lengths about 1: 2.67 : 2.58 : 1.35. Antenna: ratio of segment about 1: 1.82 : 1.41 : 0.91. Pronotum length 1.13–1.35 (mean = 1.25), width 1.13–1.84 (mean = 1.65). Hemelytra length 1.60–4.97 (mean = 4.25). Abdomen: length 2.69–3.05 (mean = 2.88), width 1.77–2.13 (mean = 1.88). Legs: fore femora: length 1.70–1.91 (mean = 1.80), width 0.35–0.42 (mean = 0.37); middle femora: length 1.60–1.77 (mean = 1.66), width 0.28–0.32 (mean = 0.29); hind femora 2.27–2.48 (mean = 2.40), width 0.15–0.21 (mean = 0.19). Fore tibiae: length 1.45–1.52 (mean = 1.48), width 0.10–0.14 (mean = 0.12); middle tibiae: length 1.55–1.63 (mean = 1.60), width 0.10; hind tibiae: length 2.84–3.05 (mean = 2.89), width 0.10.

##### Description.

Pronotum in macropterous form greatly expanded behind, a third wider than long; posterior lobe markedly arched upward. Pronotum in brachypterous form visibly wider than long, but strongly compressed on the sides between the anterior and posterior lobes. Hemelytra in macropterous forms surpassing apex of abdomen; in brachypterous forms, reaching to the base of sixth abdominal segment. Paramere with the distal area more thickened than in the other species of the complex; the blade wide, with a protuberance on the outer margin. Base of the paramere more constricted than in *Nabis paranensis*. Female genitalia with the styloid more thickened than in *Nabis paranensis*.

#### Nabis faminei

Stål, 1859

http://species-id.net/wiki/Nabis_faminei

**Figure 9.**
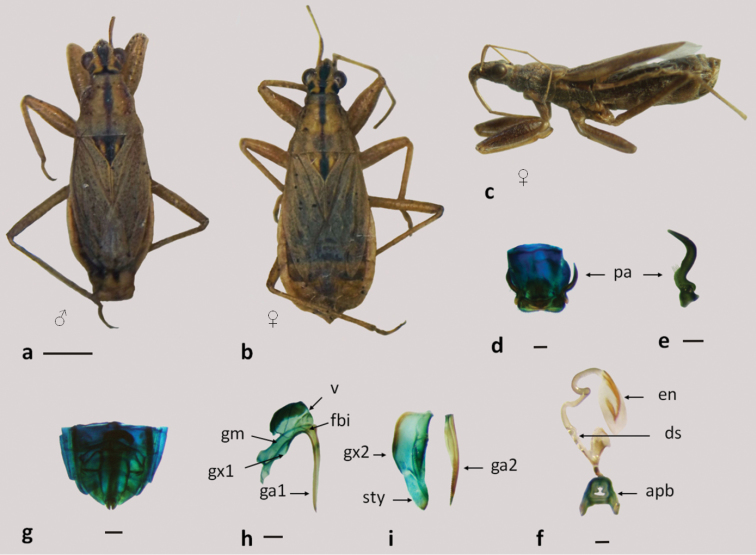
*Nabis faminei* **a–b** dorsal view **a** male **b** female **c** lateral view **d–f** male genitalia: **d** pygophore **e** paramere **f** aedeagus **g–i** female genitalia: **g** genital segment **h** first gonapophysis and gonocoxite 1 **i** second gonapophysis and gonocoxite 2. (apb: articulatory apparatus; ds: ductus seminis; en: endosoma; fbi: internal fibula; ga1 and ga2: gonapophysis 1 and 2; gm: gonangulum; gx1 and 2: gonocoxites 1 and 2, pa: paramere; sty: styloid; v: vagina). Figures **a–c** scale line 1mm; **d–i** scale line 0.2mm.

Figs 9a–i , 11c , 12c , 13d

Nabis faminei Stål 1859. Kongliga Svenska Fregattens Eugenies Resa Omkring Jorden 4: 260. Lectotype (NHRS). Reuter 1872. Öfversigt af Kongliga Svenska Vetenskaps-Akademiens Forhandlingar 29: 92. Berg 1879. Anales de la Sociedad Científica Argentina: 145. Pennington 1921. Lista de los Hemípteros de la República Argentina. Segunda parte: 26. Harris 1939. Notas del Museo de La Plata 26: 372. Prado 2008. Boletín del Museo Nacional de Historia Natural Chile 57: 44. Volpi and Coscarón 2010. Zootaxa 2513: 58.
Nabis punctipennis : Reuter 1908. Mémoires de la Société Entomologique de Belgique 15: 122. *Lectotype*: http://www2.nrm.se/en/het_nrm/f/nabis_faminei.html

##### Material examined

. BUENOS AIRES: La Plata 34°55'2.28"S, 57°57'0.47"W 1 ♀ (MLP). TIERRA DEL FUEGO: Harris det., 1 ♀ (MLP); Cabana Ruby 17–I–1988, 1 ♀ (MLP); Cabo Espíritu Santo 52°39'32.29"S, 68°36'8.83"W, 18–I–1988 4 ♂♂, 1 ♀ (MLP); Estancia La Indiana 54°20'46.48"S, 67°25'44.27"W, 17–I–1988 2 ♂♂, 1 ♀ (MLP); Lago Fagnano 17–I–1988 Leg. Molta and Lombardo col., 2 ♀♀ (MLP); Paso Garibaldi 54°41'29.80"S, 67°51'0.99"W, 17–I–1988 (MLP); Río Chico, Las Violetas 54°47'55.02"S, 68°18'29.55"W, 13–I–1988 2 ♀♀ (MLP).

##### Distribution in Argentina.

Buenos Aires: La Plata; Patagonia, Tierra del Fuego: Cabana Ruby, Cabo Espíritu Santo, Estancia La Indiana, Lago Fagnano, Paso Garibaldi, Río Chico (Las Violetas).

##### Distribution outside Argentina.

Chile.

##### Measurements.

Male (n = 3): Length 4.92–5.84 (mean = 5.26). Head: length 0.82–0.93 (mean = 0.87), width 0.82–0.86 (mean = 0.83); eye width 0.36–0.37 (mean = 0.365), interocular width 0.37. Rostrum: ratio of segment lengths about 1: 2.26 : 0.59 : 0.62. Antenna: ratio of segment about 1: 1.64 (segments III and IV missing). Pronotum length 1.01–1.16 (mean = 1.08), width 1.16–1.46 (mean = 1.28). Hemelytra length 2.44–3.79 (mean = 2.91). Abdomen: length 2.17–2.66 (mean = 2.48), width 1.50– 1.67 (mean = 1.57). Legs: fore femora: length 1.36–1.39 (mean = 1.38), width 0.39–0.41 (mean = 0.396); middle femora: length 1.25–1.27 (mean = 1.265), width 0.30–0.33 (mean = 0.31); hind femora 1.78–1.87 (mean = 1.82), width 0.17–0.18 (mean = 0.175). Fore tibiae: length 1.09–1–15 (mean = 1.12), width 0.09–0.12 (mean = 0.10); middle tibiae: length 1.06–1.17 (mean = 1.13), width 0.075–0.09 (mean = 0.08); hind tibiae: length 2.03–2.14 (mean = 2.08), width 0.06–0.07 (mean = 0.065).

Female(n = 3): Length 5.07–5.84 (mean = 5.39). Head: length 0.82–0.93 (mean = 0.88), width 0.78–0.86 (mean = 0.82); eye width 0.36–0.39 (mean = 0.37), interocular width 0.36–0.37 (mean = 0.365). Rostrum: ratio of segment lengths about 1: 2.19 : 2.43 : 1.19. Antenna: ratio of segment about 1: 1.47 : 1.41 : 1.15. Pronotum length 1.09–1.20 (mean = 1.13), width 1.27–1.42 (mean = 1.32). Hemelytra length 2.60–3.12 (mean = 2.84). Abdomen: length 2.42–2.59 (mean = 2.52), width 1.80–1.95 (mean = 1.86). Legs: fore femora: length 1.09–1.60 (mean = 1.35), width 0.36–0.42 (mean = 0.38); middle femora: length 1.21–1.51 (mean = 1.33), width 0.21–0.30 (mean = 0.26); hind femora 1.75–2.24 (mean = 1.94), width 0.18. Fore tibiae: length 1.18–1.33 (mean = 1.24), width 0.09–0.12 (mean = 0.11); middle tibiae: length 1.15–1.33 (mean = 1.23), width 0.09–0.10 (mean = 0.093); hind tibiae: length 2.12–2.39 (mean = 2.21), width 0.075–0.10 (mean = 0.088).

##### Description.

Similar to *Nabis punctipennis* but with the body more robust, the eyes larger, and the antennae and legs shorter. Length of antennal segment II hardly equal to the width of the head across eyes. Anterior lobe of pronotum arcuate, strongly raised above the collar. Hemelytra exposing part of the abdomen. Legs robust, anterior femora thickened. Paramere blade thin, with apex pointed. Female genitalia with thick androbust styloid.

#### Nabis punctipennis

Blanchard, 1852

http://species-id.net/wiki/Nabis_punctipennis

**Figure 10.**
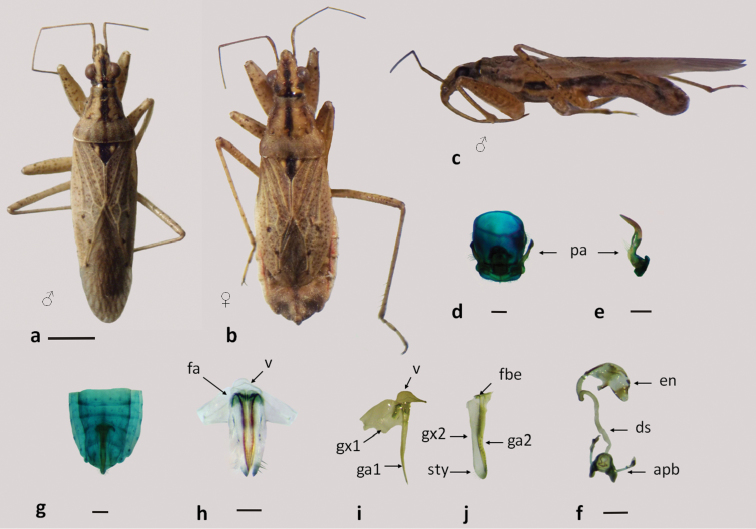
*Nabis punctipennis* **a–b** dorsal view **c** lateral view **d–f** male genitalia: **d** pygophore **e** paramere **f** aedeagus **g–j** female genitalia: **g–h** genital segment **i** first gonapophysis and gonocoxite 1 **j** second gonapophysis and gonocoxite 2. (apb: articulatory apparatus; ds: ductus seminis; en: endosoma; fa: anterior fibula; fbe: external fibula; ga1 and ga2: gonapophysis 1 and 2; gx1 and 2: gonocoxites 1 and 2, pa: paramere; sty: styloid; v: vagina). Figures **a–c** scale line 1mm; **d–j** scale line 0.2mm.

**Figure 11.**
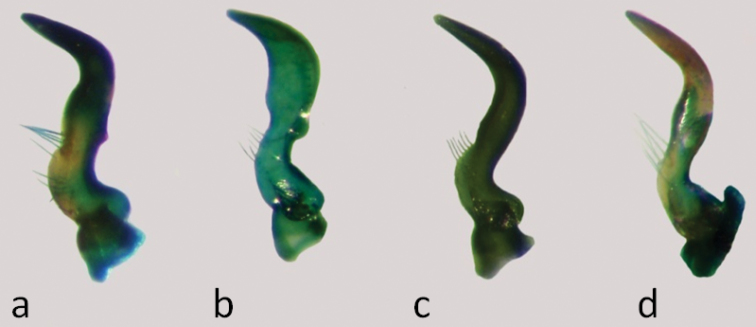
Parameres: **a** *Nabis paranensis* **b** *Nabis argentinus* **c** *Nabis faminei* **d** *Nabis punctipennis*.

**Figure 12.**
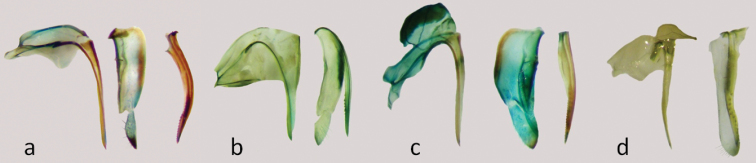
Genital segments of female: **a** *Nabis paranensis* **b** *Nabis argentinus* **c** *Nabis faminei* **d** *Nabis punctipennis*.

**Figure 13.**
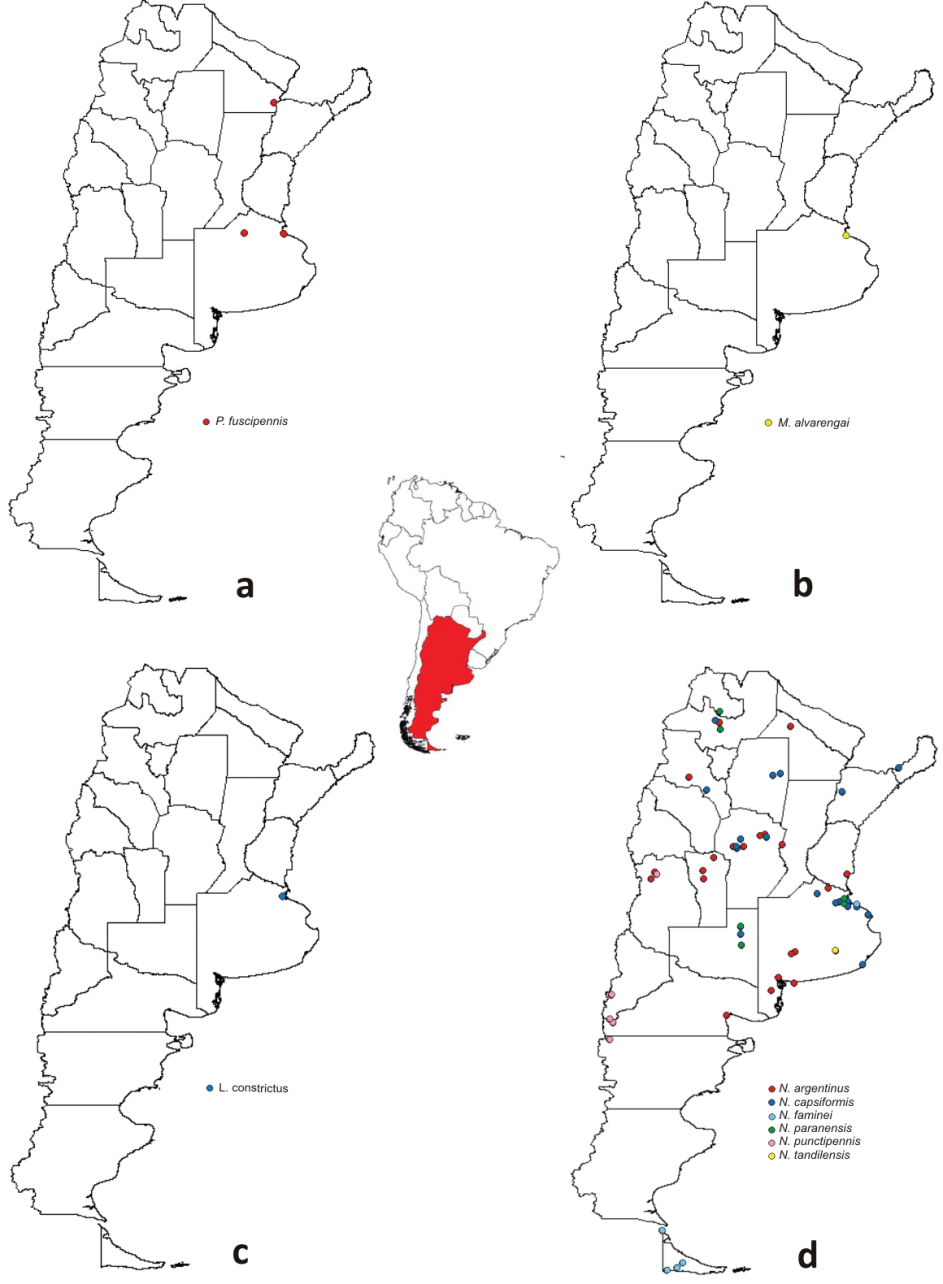
Geographical distribution of species of Nabidae in Argentina: **a** *Pagasa* **b** *Metatropiphorus* **c** *Lasiomerus* **d** *Nabis*.

Figs 10a–j , 11d , 12d , 13d

Nabis punctipennis Blanchard 1852. In: Gay C (Ed) Historia Física y Política de Chile 7: 161. Signoret 1863. Annales de la Société Entomologique de France 3: 577. Reuter 1872. Öfversigt af Kongliga Svenska Vetenskaps-Akademiens Forhandlingar29: 90. Harris 1939. Notas del Museo de La Plata 26: 370–371. Prado 2008. Boletín del Museo Nacional de Historia Natural Chile 57: 44. Volpi and Coscarón 2010. Zootaxa 2513: 59.
Nabis parvulus Reuter 1872. Öfversigt af Kongliga Svenska Vetenskaps-Akademiens Forhandlingar29: 90–91. Synonymized by Reuter 1908: 123.
Coriscus punctipennis Stål 1873. Kongliga Svenska Vetenskaps-Akademiens Handlingar 11: 114.
Reduviolus punctipennis Reuter 1908. Mémoires de la Société Entomologique de Belgique 15: 122 (in part).

##### Material examined.

CHUBUT: El Hoyo 42°3'51.98"S, 71°31'11.29"W, 21–I–1965 A Kovacs col., Kerzhner det., 1987 1 ♂, 1 ♀ (AMNH). MENDOZA: Jensen-Haarup det., 1 ♀ (MHND); Chacras de Coria 32°58'51.27"S, 68°52'36.84"W Jensen-Haarup, 1 ♀ (MHND). NEUQUÉN: Huechulaufquen 39°46'4.95"S, 71°22'14.02"W, 23–II–1942 M. Biraben col., 1 ♀ (MLP); Nahuel Huapi, Isla Victoria 40°55'59.99"S, 71°33'0.04"W, 1–XI–1969 Bosq col., 1 ♂ (MLP). RIO NEGRO: San Carlos de Bariloche 41°8'57.44"S, 71°18'4.57"W, 2–IV–1964 A Kovacs col., Kerzhner det., 1987 1 ♂, 1 ♀ (AMNH).

##### Distribution in Argentina.

Chubut: El Hoyo; Mendoza: Chacras de Coria; Neuquén: Huechulaufquen, Nahuel Huapi: Isla Victoria; Río Negro: San Carlos de Bariloche.

##### Distribution outside Argentina.

Chile: Colina, Osorno, Puerto Varas. Continental Chile and Archipiélago Juan Fernández.

##### Measurements.

Male(n = 2): Length 5.26–6.24 (mean = 5.75). Head: length 0.86–0.88 (mean = 0.87), width 0.71–0.86 (mean = 0.78); eye width 0.33–0.39 (mean = 0.36), interocular width 0.37–0.39 (mean = 0.38). Rostrum: ratio of segment lengths about 1: 1.91 : 2.08 : 0.91. Antenna: ratio of segment about 1: 1.74: 1.44: 1.33. Pronotum length 1.16–1.23 (mean = 1.19), width 1.27–1.42 (mean = 1.34). Hemelytra length 2.63–4.11 (mean = 3.37). Abdomen: length 2.25–2.41 (mean = 2.33), width 1.27– 1.39 (mean = 1.33). Legs: fore femora: length 1.63–1.69 (mean = 1.66), width 0.35–0.37 (mean = 0.36); middle femora: length 1.46–1.49 (mean = 1.475), width 0.28–0.30 (mean = 0.29); hind femora 2.05–2.10 (mean = 2.07), width 0.14–0.15 (mean = 0.145). Fore tibiae: length 1.31–1–42 (mean = 1.36), width 0.10–0.11 (mean = 0.105); middle tibiae: length 1.35, width 0.09–0.10 (mean = 0.095); hind tibiae: length 2.36–2.48 (mean = 2.42), width 0.07–0.10 (mean = 0.085).

Female(n = 5): Length 4.92–6.67 (mean = 6.06). Head: length 0.81–1.06 (mean = 0.91), width 0.78–0.92 (mean = 0.84); eye width 0.33–0.42 (mean = 0.37), interocular width 0.31–0.40 (mean = 0.35). Rostrum: ratio of segment lengths about 1: 2.30 : 2.33 : 1.24. Antenna: ratio of segment about 1: 1.61 : 1.33 : 1.15. Pronotum length 1.12–1.35 (mean = 1.24), width 1.20–1.77 (mean = 1.52). Hemelytra length 2.44–4.61 (mean = 3.68). Abdomen: length 2.48–3.26 (mean = 2.83), width 1.57–2.13 (mean = 1.78). Legs: fore femora: length 1.51–1.61 (mean = 1.56), width 0.39–0.42 (mean = 0.40); middle femora: length 0.45–1.68 (mean = 1.55), width 0.26–0.35 (mean = 0.30); hind femora 1.87–2.60 (mean = 2.21), width 0.15–0.21 (mean = 0.17). Fore tibiae: length 1.31–1.42 (mean = 1.35), width 0.07–0.11 (mean = 0.10); middle tibiae: length 1.27–1.60 (mean = 1.42), width 0.07–0.11 (mean = 0.09); hind tibiae: length 1.81–2.77 (mean = 2.40), width 0.07–0.10 (mean = 0.08).

##### Description.

Length of antennal segment II slightly longer than the width of the head across eyes. Hemelytra in macropterous form surpassing the apex of abdomen, in brachypterous form reaching eight segment. Paramere very similar to *Nabis faminei*, but with the blade slightly more widened and the base more constricted. Female genitalia with the styloid less thickened than in *Nabis faminei*.

## Supplementary Material

XML Treatment for Pagasa

XML Treatment for Pagasa (Lampropagasa)

XML Treatment for Pagasa fuscipennis

XML Treatment for Pagasa (Pagasa)

XML Treatment for Pagasa costalis

XML Treatment for Pagasa signatipennis

XML Treatment for Metatropiphorus

XML Treatment for Metatropiphorus alvarengai

XML Treatment for Lasiomerus

XML Treatment for Lasiomerus constrictus

XML Treatment for Hoplistoscelis

XML Treatment for Hoplistoscelis sordida

XML Treatment for Nabis

XML Treatment for Nabis tandilensis

XML Treatment for Tropiconabis

XML Treatment for Nabis capsiformis

XML Treatment for Nabis seticrus

XML Treatment for Nabis roripes

XML Treatment for Nabis paranensis

XML Treatment for Nabis argentinus

XML Treatment for Nabis faminei

XML Treatment for Nabis punctipennis
